# Effects of nitrogen application on phytochemical component levels and anticancer and antioxidant activities of *Allium fistulosum*

**DOI:** 10.7717/peerj.11706

**Published:** 2021-06-24

**Authors:** Chen Zhao, Zhongjian Wang, Rongzong Cui, Le Su, Xin Sun, Orlando Borras-Hidalgo, Kunlun Li, Jianlin Wei, Qiulin Yue, Lin Zhao

**Affiliations:** 1 State Key Laboratory of Biobased Material and Green Papermaking, Shandong Provincial Key Lab. of Microbial Engineering, Qilu University of Technology (Shandong Academy of Sciences), Jinan, China; 2 Institute of Agricultural Resources and Environment, Shandong Academy of Agricultural Sciences, Jinan, China; 3 Jinan Hangchen Biotechnology Co., Ltd., Jinan, China; 4 Institute of Applied Ecology, Chinese Academy of Sciences, Shenyang, China

**Keywords:** *A. fistulosum*, Nitrogen level, Phenolics, Flavonoids, Antioxidant, Anticancer

## Abstract

**Background:**

*Allium fistulosum* L. has good nutritional value and is cultivated worldwide as an efficacious traditional medicinal plant. Its biological activities are attributable to its phytochemicals. Nitrogen is an essential nutrient for plant growth and development; however, the effect of nitrogen levels on the level of active components in this species is not well understood.

**Methods:**

In this study, using urea fertilizer, we investigated the effects of different nitrogen levels (N0, N1, and N2 at 0, 130, and 260 kg/ha, respectively) on the phytochemical constituents , and antioxidant and anticancer properties of *A. fistulosum*.

**Results:**

The results suggested that nitrogen fertilizers have a significant effect on the level of total phenols and flavonoids. The analysis of the antioxidant capacity revealed that the lowest IC_50_ values corresponded to plants treated with the highest nitrogen concentration. Anticancer activity was investigated against cancer cell lines (HeLa and HepG2), and the extracts of *A. fistulosum* treated with a high nitrogen level showed the highest antiproliferative effect. Collectively, our results suggest that nitrogen fertilizer application enhanced the quality of *A. fistulosum*, particularly its health benefits.

## Introduction

*Allium fistulosum* L. (Welsh onion) is extensively cultivated in China, Japan, and Korea. It is an indispensable ingredient for flavoring many Asian dishes. Moreover, it is considered a good source of nutrients and is used as a traditional medicine. Several studies have described the benefits of *A. fistulosum* for human health, including its antioxidant (Zuo et al., 2018), antibacterial (Chang et al., 2016), anticancer (Pan, Zheng & Ho, 2018), antihypercholesterolemic (Choi et al., 2017), anti-obesity (Sung et al., 2018), and anti-inflammatory (Wang et al., 2013) properties. The active compounds of *A. fistulosum* contribute to its various biological activities. For example, the antioxidant capacity of *A. fistulosum* is highly correlated with its total phenolic content, whereas its antibacterial activity is contributable to allicin (Chang et al., 2016). Flavonoids in Welsh onion, including quercetins and quercetin glycosides, have been reported to be associated with its anticancer and antioxidant properties (Sun et al., 2002; Pan, Zheng & Ho, 2018).

Nitrogen is one of the most important nutrients required by plants. Nitrogen fertilizers influence the productivity and yield of various plants worldwide, including *Astragalus mongolica* (Wang et al., 2020), rice (Tang et al., 2019), and wheat (Li et al., 2019). Nitrogen level might also influence plant secondary metabolism. For example, nitrogen application increased the essential oil yield of patchouli plants compared with that in plants grown without nitrogen (Singh, Sharma & Ramesh, 2002). Medium levels of nitrogen and phosphorus application increased saikosaponin production in the roots of *Bupleurum chinense* compared with low levels of application (Zhu et al., 2009). However, the level of some active compounds in *Rheum tanguticum* was not influenced by nitrogen level (Xiong et al., 2019). In *Allium* species, the effects of nitrogen on growth and yield have been well described (Brewster & Butler, 1989; Neeraja et al., 2001; Gebretsadik & Dechassa, 2018); however, not much is known about the influence of nitrogen on the active compounds. Two main groups of components have been proposed as candidates responsible for the beneficial effects of onions: the flavor precursors, namely S-alk(en)yl cysteine sulfoxides (ACSOs), and the flavonoids (Griffiths et al., 2002). A previous study showed that nitrogen fertilization level had almost no effect on onion quercetin content (Mogren, Olsson & Gertsson, 2006); in that study, nitrogen was applied at a slightly lower level than that used in conventional production. The objective of the present study was to determine the influence of nitrogen on the content of beneficial compounds. To this end, we determined the phytochemical constituents, as well as the antioxidant and anticancer properties of Welsh onion, using fertilizers with different nitrogen concentrations.

## Materials & Methods

### Plant materials and experimental area

*Allium fistulosum* seedlings were obtained from Shandong Academy of Agricultural Sciences, Shandong Province, China, and the seedlings were grown in a field in Zhangqiu District, Jinan City, Shandong Province, China (36°72′N, 117°53′E). The experimental area has a sub-humid continental monsoon climate, with a mean annual temperature of 12.6 °C. The soil type of the area is classified as cinnamon soil. The initial nitrogen concentration in the soil was 80.67 mg kg^−1^.

### Experimental design

A randomized block experiment with three treatments and five replicates was conducted. Plots without the application of urea were regarded as control plots (N0). Urea was used as the N-source fertilizer, and applied at two levels: half level of nitrogen (130 kg/ha) and total level of nitrogen (260 kg/ha), represented here as N1 and N2, respectively. The fertilizer was applied four times: on June 25, August 14, August 27, and September 9, 2017. Twenty percent of the nitrogen fertilizer was applied as base fertilizer before the planting of Welsh onions in the end of June. The nitrogen fertilizer was applied according to the application habit in the Welsh onion planting area. Fertilization was carried out according to nutrient requirements in different growth periods of Welsh onion. The experimental field included 15 plots, each 2 m apart and covering an area of 0.8 m^2^. In each replicate of the three fertilizer levels, 15 plants were randomly selected for harvesting on October 31, 2017, and further analyses were performed.

### Chemicals used

Gallic acid, quercetin, kaempferol, quercetin-3-*β*-D-glucoside, 2,2-diphenyl-1-picrylhydrazyl (DPPH), 2,2′-azino-bis(3-ethylbenzothiazoline-6-sulfonic acid (ABTS), 3-(4,5-dimethylthiazole-2-yl)-2,5-diphenyltetrazolium bromide (MTT), ascorbic acid, and 5-fluorouracil were acquired from Sigma-Aldrich (St. Louis, MO, USA). Folin–Ciocalteu reagent was acquired from Sinopharm Chemical Reagent Co., Ltd. (Shanghai, China). Other chemicals were of analytical grade.

### Sample extraction

The stems of Welsh onion were cut, frozen at −80 °C, lyophilized, and then reduced to a powdered form. The samples were extracted as follows: 1 g of lyophilized powder was stirred in nine mL of aqueous ethanol (70%) and sonicated for 1 h. The mixtures were filtered, and the supernatants were concentrated under reduced pressure. The extracts were then lyophilized and stored at −20 °C until further analyses.

### Determination of total phenolic content

The total phenolic content of the extracts was determined using a modified Folin–Ciocalteu method (Chang et al., 2016). Briefly, one mL of diluted extract was mixed with one mL of 1 M Folin–Ciocalteu reagent and agitated for 2 min, followed by the addition of two mL of Na_2_CO_3_ (20% w/v). After 8 min of incubation, the mixture was centrifuged at 10, 000 × *g* for 10 min. The absorbance of the supernatant was measured at 730 nm using a UV-Vis spectrophotometer (Hitachi U 2910, Kyoto, Japan), and a calibration curve for gallic acid was obtained. The total phenolic content is expressed as milligrams of gallic acid equivalents per 100 g of dry material (mg GAE/100 g).

### High-performance liquid chromatography (HPLC) of flavonoids

High-performance liquid chromatography was conducted according to a modified method of Dai & Row (2019), using a Shimadzu LC-20A HPLC with a UV/VIS detector set at a wavelength of 285 nm. The column used was an Agilent C18 column (250 mm ×4.6 mm, i.d. 5 µm) coupled to an Agilent C18 guard column (4.6 mm ×12.5 mm, i.d. 5 µm). The mobile phase was 0.2% acetic acid (in HPLC grade water) and acetonitrile at a ratio of 65:35 (v/v). The injection volume was 20 µL, and flow rate was 1.0 mL/min. At least three separate injections were prepared for each sample. The standard flavonoid compounds used were quercetin, kaempferol, and quercetin-3-*β*-D-glucoside. Serial dilutions ranged from 10 to 200 µg/mL for each standard.

### ABTS radical cation scavenging assay

The ABTS assay was conducted according to the method of Assefa et al. (2019), with some modifications. In brief, the ABTS radical cation was generated by mixing an ABTS stock solution (7 mM) with potassium persulfate solution (2.4 mM) at a ratio of 1:1. The mixture was incubated overnight at 25 °C in the dark. The ABTS radical cation solution was further diluted with distilled water to obtain an absorbance of 0.7 ± 0.02 at 734 nm. Gallic acid was used as the standard, and ethanol was used as the control. After adding 0.1 mL of the extracts (at concentrations of 0.1, 0.08, 0.04, 0.02, and 0.01 mg/mL) or standard to 2.9 mL of diluted ABTS radical cation solution, and allowing it to stand for 8 min, the absorbance was measured using a UV-Vis spectrophotometer (Hitachi U 2910; Kyoto, Japan). The percentage of radical scavenging capacity was calculated as follows: scavenging activity (%) = [1 − (A_1_ − A_2_)/A_0_] ×100, where *A*_0_ is the absorbance of the control, *A*_1_ is absorbance of the test solution of sample extracts, and *A*_2_ is the absorbance of the sample blank. The IC_50_ value, which represents the concentration of extract required for 50% inhibition of ABTS radicals, was determined next.

### DPPH radical scavenging capacity analysis

DPPH radical scavenging capacity of the extracts was determined using the method described previously (Chang et al., 2016), with minor modifications. In brief, two mL of 0.1 mM freshly prepared DPPH ethanolic solution was added to one mL of samples at concentrations of 0.1, 0.08, 0.04, 0.02, and 0.01 mg/mL or standard. The mixture was agitated vigorously and placed in the dark for 30 min at 25 °C. Ascorbic acid was used as the standard antioxidant, and ethanol was used as the control. Absorbance of the solution was measured at 517 nm using a UV-Vis spectrophotometer (Hitachi U 2910). The percentage of DPPH radical scavenging capacity was calculated as follows: scavenging activity (%) = [(A_0_ − A_1_) /A_0_] ×100, where *A*_0_ is the absorbance of the control, and *A*_1_ is the absorbance of the sample. The IC_50_ value was calculated using linear regression analysis.

### Anticancer activity assay

HeLa and HepG2 cells were acquired from the Cell Bank of the Chinese Academy of Sciences. The cells were cultured in DMEM supplemented with 10% fetal bovine serum. Both cell lines were maintained at 37 °C with 5% CO_2_. The cells were seeded into 96-well flat-bottom plates and cultured for 24 h. All samples were dissolved in DMSO and diluted to various concentrations (100, 200, and 400 µg/mL), with 0.1% (v/v) DMSO as the negative control. 5-Fluorouracil (10 µg/mL) was utilized as the positive control. Approximately 48 h after treatment with *A. fistulosum* extracts or the control, the MTT assay was conducted to determine cell viability. The absorbance was measured at 570 nm using a microplate reader (MD SpectraMax i3x; San Jose, CA, USA). The results, expressed as a percentage, were compared with those of the control to determine relative cell viability. All experiments were conducted in triplicate.

### Statistical analysis

The data are expressed as mean ± SD. All results were analyzed using the one-way ANOVA with Bonferroni correction to investigate the differences across the groups. *P*-values lower than 0.05 were considered statistically significant. All analyses were performed using the Origin software (version 9.1).

## Results

### Effect of nitrogen level on total phenolic content

The total phenolic content in the extracts of *A. fistulosum*, after treatment with different levels of N, is shown in Table 1. Among the different Welsh onion extracts, the highest phenolic content was observed in the N2 treatment group (22.66 ±0.50 mg GAE/100 g dry weight). The total phenolic content in the N0 group was the lowest, whereas that in the N1 group was slightly higher than that in the N0 group. However, the difference between the groups was not significant.

**Table 1:** Total phenolic content of Welsh onion extracts after treatment with different nitrogen levels.

Treatment	Total phenolic content, mg GAE/100 g dry weight
N0	17.98 ± 0.98^b^
N1	19.43 ± 0.47^ab^
N2	22.66 ± 0.50^a^

**Notes.**

Data are represented as mean ± SD. *P* < 0.05 is indicated by different letters.

### Effect of nitrogen level on the flavonoid content

**Figure 1:**
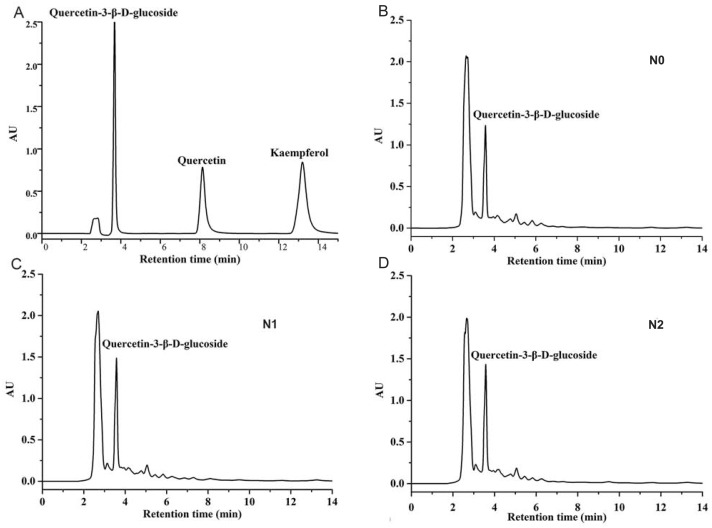
HPLC chromatograms of quercetin, kaempferol, quercetin-3- *β*-D-glucoside standards (A) and Welsh onion extracts at three different nitrogen levels (B–D).

In the present study, three types of flavonoids were detected, by HPLC, in *A. fistulosum* treated with different levels of nitrogen fertilizers (Fig. 1). Quercetin and kaempferol were not detected in any sample (Table 2). The level of quercetin-3-*β*-D-glucoside was the highest in samples from the N1 treatment group (760.01 ± 86.52 mg/100 g dry weight), and it was significantly higher than that in samples from the N0 treatment group. Moreover, the quercetin-3-*β*-D-glucoside level was slightly lower in samples from the N2 group than in samples from the N1 group; however, the difference was not significant.

**Table 2:** Quantification of flavonoid compounds in Welsh onion extracts by high-performance liquid chromatography.

Treatment	Flavonoid compound content, mg/100 g dry weight		
	Quercetin	Kaempferol	Quercetin-3-*β*- D-glucoside
N0	ND	ND	159.70 ± 16.38^b^
N1	ND	ND	760.01 ± 86.52^a^
N2	ND	ND	468.97 ± 64.43^ab^

**Notes.**

ND, not detected. Data are represented as mean ± SD. Different letters within the same column indicate a significant difference at *P* < 0.05.

### Effect of nitrogen level on antioxidant activities of the extracts

Extracts of plants from the N2 treatment group presented the highest ABTS scavenging ability followed by those from the N1 and N0 groups (Table 3). The same trend was observed for DPPH scavenging ability (Table 4). Extracts from the N2 group had the lowest IC_50_ value of 58.79 ±  1.73 µg/mL for ABTS and 50.74 ±  0.53 µg/mL for DPPH scavenging. A lower IC_50_ value indicates a higher antioxidant activity. The findings revealed that extracts of plants treated with higher levels of nitrogen exhibited a higher antioxidant effect.

**Table 3:** ABTS scavenging capacity of Welsh onion (0.1 mg/mL) and the IC_50_ value at different nitrogen levels.

Treatment	ABTS radical scavenging ability (%)	IC_50_ value (µg/mL)
N0	57.44 ± 0.45^c^	78.27 ± 2.9^a^
N1	62.19 ± 0.23^b^	69.31 ± 2.89^a^
N2	80.99 ± 1.33^a^	58.79 ± 1.73^a^
Ascorbic acid	83.10 ± 1^a^	51.04 ± 0.45^b^

**Notes.**

Data are represented as mean ± SD. Different letters within the same column indicate a significant difference at *P* < 0.05.

**Table 4:** DPPH scavenging capacity of Welsh onion (0.1 mg/mL) and IC_50_ value at different nitrogen levels.

Treatment	DPPH radical scavenging ability (%)	IC_50_ value (µg/mL)
N0	59.45 ± 0.24^b^	89.57 ± 1.11^a^
N1	68.26 ± 1.15^b^	67.11 ± 1.49^b^
N2	82.12 ± 2.68^a^	50.74 ± 0.53^c^
Gallic acid	87.41 ± 0.57^a^	45.72 ± 2.86^c^

**Notes.**

Data are represented as mean ± SD. Different letters within the same column indicate a significant difference at *P* < 0.05.

### Effect of nitrogen level on the anticancer activities of the extracts

**Figure 2:**
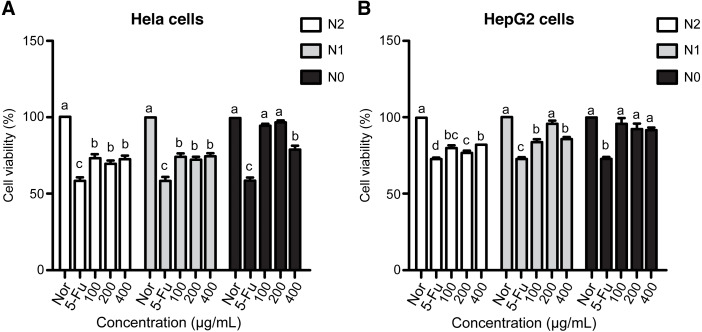
Anticancer activities of Welsh onion under N0, N1, and N2 treatments on HeLa (A) and HepG2 (B) cancer cell lines. Data are represented as mean ±  SD. Means with different superscript letters (a–d) in the same row indicate a significant difference at *P* < 0.05.

5-Fu considerably inhibited cancer cell growth even at very low concentrations. The viability of HeLa and HepG2 cell lines treated with 10 µg/mL 5-Fu was 58.94% and 73.20%, respectively (Fig. 2). Compared with the reference drug, the extracts of Welsh onion exhibited moderate cytotoxicity against cell lines. The viability of HeLa and HepG2 cell lines exhibited a similar trend among the three treatments. Concentration difference of the samples had little influence on cell growth. Samples from the N1 and N2 groups at concentrations of 100, 200, and 400 µg/mL had significantly higher anticancer activity toward the two cancer cell lines than the negative control samples. However, this phenomenon was not observed in samples from the N0 group. Three concentrations of the N0 group samples exhibited weak cytotoxicity against HepG2 cell lines, whereas a similar behavior was observed at concentrations of 100 and 200 µg/mL in HeLa cell lines. Interestingly, the viability of HeLa cell lines treated with the N0 group sample at 400 µg/mL was significantly lower than that of cells treated with normal control samples. Moreover, samples from the N2 group had a higher antiproliferative effect than those from the N1 group, although the difference was not significant (Fig. 2).

## Discussion

Genetic and environmental factors are known to influence the composition of nutritional compounds and quality of food (Tomás-Barberán & Espín, 2001). Determination of active compounds in the samples of interest is an important step for estimating the optimum level of nitrogen fertilization in *A. fistulosum* cultivation. The antioxidant activity of *Allium* species has been attributed to a variety of compounds, among which polyphenols are the most important (Nencini et al., 2007). To date, various studies have investigated the total phenolic content and relative activities of these compounds in different plant species (Kujala et al., 2000; Abdelrahman et al., 2019; Assefa et al., 2019). Studies have analyzed the phenolic content of onions among different cultivars (Yang et al., 2004; Chang et al., 2016; Metrani et al., 2020); however, information on how nitrogen fertilizers affect the phenolic content is scarce. In the present study, we determined the total phenolic content in the extracts of *A. fistulosum* after treatment with different levels of N. The accumulation of polyphenols was the highest under the N2 treatment, although there was no significant difference in the phenolic content between the N1 and N2 treatment groups. On the basis of the results of this study, we conclude that Welsh onions with a high phenolic content can be grown using the N1 fertilization level.

Previous studies have shown that certain flavonoids exhibit anti-inflammatory, antihypercholesterolemic, anticancer, and antioxidant properties (Slimestad, Fossen & Vågen, 2007; Lee & Mitchell, 2012; Kiokias & Varzakas, 2014). Among them, quercetins and quercetin glycosides have the highest activities (Pandey et al., 2016). Onion has been reported to contain a wide range of quercetin and kaempferol conjugates in different proportions (Bilyk, Cooper & Saper, 1984; Leighton et al., 1992); therefore, in the present study, quercetin, kaempferol, and quercetin-3- *β*-D-glucoside were considered. Quercetin and kaempferol were not detected in any sample, whereas the highest content of quercetin-3- *β*-D-glucoside was detected in the samples from the N1 treatment group. Reportedly, the content of free quercetin is low in most cases, whereas quercetin derivatives are reported as the main flavonols in onion cultivars (Slimestad, Fossen & Vågen, 2007), mainly as glucosidase. It has been reported that normal nitrogen levels promote the biosynthesis of flavonol glycosides through gene regulation and the accumulation of substrate carbohydrates in *Camellia sinensis*, whereas nitrogen deficiency and excess nitrogen have inhibitory effects (Dong et al., 2019). A similar trend for the content of quercetin-3- *β*-D-glucoside was observed in Welsh onion, and the mechanisms involved need further investigation. In addition, 260 and 130 kg/ha nitrogen did not lead to differences in the flavonoid content. Mogren et al. found that the quercetin content was not significantly different after treatment with high or low levels of nitrogen fertilizers (Mogren, Olsson & Gertsson, 2006); the same results were obtained in the present study as well. We concluded that nitrogen may be able to increase the flavonoid content over a certain concentration range, beyond which no further flavonoid accumulation can be induced. However, this finding has to be validated in future studies.

*Allium* species exhibit different antioxidant activities under various conditions. Previous studies have evaluated the antioxidant activities of different varieties of *Allium* species using different extraction methods (Štajner et al., 2008; Huang et al., 2009; Chang et al., 2013). In the present study, the antioxidant activities of *A. fistulosum* extracts in 70% ethanol solution were evaluated after treatment with different nitrogen levels. The N2 treatment group, which showed the best antioxidant characteristics, had the highest total phenolic content. A positive correlation between total phenolic content and antioxidant activity had been demonstrated in previous studies (Sellappan & Akoh, 2002; Sun et al., 2002), and the results of our current study are in accordance with these findings. This indicates that phenolic compounds maybe the major contributor to the antioxidant activity of Welsh onion. Optimum N fertilization may result in high total phenolic level and antioxidant effects of *Allium* species.

The phytochemicals present in the extracts of *A. fistulosum* also contribute to its anticancer activity. The anticancer effects of quercetin glucosides and polyphenols have been demonstrated previously (Sellappan & Akoh, 2002; Sun et al., 2002; Li et al., 2014). The antiproliferative activity of *A. fistulosum* extracts exhibited similar pattern against HeLa and HepG2 cancer cell lines. The results indicated the influence of nitrogen level on the anticancer activity of Welsh onion; a higher antiproliferative effect was detected at higher nitrogen fertilization levels. As the difference between the N2 and N1 groups was insignificant and considering the cost of fertilization, the application of nitrogen fertilizer at the N1 level (130 kg/ha) may be considered sufficient to stimulate the growth of *A. fistulosum*. Moreover, samples from the N0 group and the N2 and N1 groups exhibited similar anticancer activity against HeLa cells. It seems that a specific phenolic compound or a class of phenolics may contribute to the antiproliferative effect in a dose-dependent manner. At the concentration of 400 µg/mL, the active phytochemicals in the N0 group may be comparable with those in the N2 and N1 groups, resulting in a lower viability than the other two concentrations.

The findings of this study suggest that the level of nitrogen fertilization has a significant effect on the accumulation of active metabolites in Welsh onion, thereby influencing the quality and bioactivity of the phytochemicals present in the species. Knowledge of the differences in the phytochemical profiles of *A. fistulosum* in response to different levels of nitrogen supply may be of importance to determine the optimum amount of nitrogen fertilizer to be used. Furthermore, the extracts of Welsh onion grown under appropriate nitrogen levels can be a good source of natural antioxidants and used in the development of food preservatives. Further research elucidating the biosynthesis pathway of the phytochemicals would further our understanding of the effects of nitrogen on the quality of Welsh onion.

## Conclusions

In this study, we determined the effects of nitrogen levels on the active components and biological activities of *A. fistulosum*. The representative and dominant phytochemicals detected in the extracts of Welsh onion were phenolic compounds and flavonoids. We observed differences in the antioxidant and anticancer activities of *A. fistulosum* extracts based on their treatment with different nitrogen concentrations. Collectively, the findings of this study suggest that nitrogen fertilizers exert notable effects on the levels of active compounds in Welsh onion, showing a particularly strong influence on its antioxidant and anticancer activities.

##  Supplemental Information

10.7717/peerj.11706/supp-1 Supplemental Information 1 Raw data for phytochemical component levels and anticancer and antioxidant activities

## Additional Information and Declarations

### Data Availability

The following information was supplied regarding data availability: The raw data are available in the Supplementary File.
